# A New Low-Temperature Electrochemical Hydrocarbon and NO_x_ Sensor

**DOI:** 10.3390/s17122759

**Published:** 2017-11-29

**Authors:** Praveen Kumar Sekhar, Zachary Moore, Shyam Aravamudhan, Ajit Khosla

**Affiliations:** 1 Nanomaterials and Sensors Laboratory, School of Engineering and Computer Science, Washington State University Vancouver, Vancouver, WA 98686, USA; zachary.a.moore@wsu.edu; 2 Joint School of Nanoscience and Nanoengineering, North Carolina A & T State University, Greensboro, NC 27401, USA; saravamu@ncat.edu; 3 Faculty of Engineering, Yamagata University, Yonezawa, Yamagata 992-8510, Japan; khosla@gmail.com

**Keywords:** electrochemical, YSZ, STO, oxygen ion conductivity, NO_x_, hydrocarbon

## Abstract

In this article, a new investigation on a low-temperature electrochemical hydrocarbon and NO_x_ sensor is presented. Based on the mixed-potential-based sensing scheme, the sensor is constructed using platinum and metal oxide electrodes, along with an Yttria-Stabilized Zirconia (YSZ)/Strontium Titanate (SrTiO_3_) thin-film electrolyte. Unlike traditional mixed-potential sensors which operate at higher temperatures (>400 °C), this potentiometric sensor operates at 200 °C with dominant hydrocarbon (HC) and NO_x_ response in the open-circuit and biased modes, respectively. The possible low-temperature operation of the sensor is speculated to be primarily due to the enhanced oxygen ion conductivity of the electrolyte, which may be attributed to the space charge effect, epitaxial strain, and atomic reconstruction at the interface of the YSZ/STO thin film. The response and recovery time for the NO_x_ sensor are found to be 7 s and 8 s, respectively. The sensor exhibited stable response even after 120 days of testing, with an 11.4% decrease in HC response and a 3.3% decrease in NO_x_ response.

## 1. Introduction

Electrochemical gas sensors [1,2,3,4,5,6] offer a portable, selective, sensitive, rapid, and compact solution to detect analytes for various applications including clean coal power plants, automotive emissions, hydrogen safety, air quality, and breathe analysis to identify chronic disorders. Among the various detection methodologies in electrochemical (EC) gas sensors, the mixed-potential-based scheme [7] possesses some unique features such as (a) simple device architecture; (b) compactness, to fit into critical areas; (c) fast response; and (d) direct voltage read-out, circumventing the need for additional conditioning circuitry.

Mixed-potential electrochemical sensors fabricated using well-established commercial manufacturing methods present a promising avenue to enable the widespread utilization of gas sensors. These devices are fundamentally simple and robust, owing to their close relationship to the ubiquitous automotive Lambda sensor. The mixed-potential sensors develop a non-Nernstian potential due to differences in the redox kinetics of various gas species at each electrode/electrolyte gas interface in the presence of oxygen [8,9,10,11]. The difference in the steady-state redox reaction rates between the fast and slow electrodes gives rise to the measured mixed-potential response. A stable sensor design incorporates dense electrodes and a porous electrolyte, using a high-temperature ceramic co-fire (HTCC) approach [12]. The use of dense electrodes (a) minimizes deleterious heterogeneous catalysis (leading to reduced sensitivity); and (b) increases the morphological stability, yielding a robust electrochemical interface, thereby increasing lifetime durability. The downside of mixed-potential sensors is the high power consumption due to the high temperature of sensor operation. While high-temperature operation is suitable for certain applications such as emissions control, the majority of the sensor applications demand ultra-low power consumption. In addition, to realize low-power operation, the sensor should be operable at low temperature without compromising on its performance. The high-temperature operation is necessary mainly due to the presence of the well-known solid electrolyte Yttria-Stabilized Zirconia (YSZ) in the sensor fabrication.

Yttria-Stabilized Zirconia (YSZ) is an oxygen ion conductor, where oxygen vacancies are introduced by aliovalent substitution of Zr by Y. The substitution of Zr in a 4+ oxidation state with Y^3+^ produces an oxygen vacancy for every two Y atoms. As mentioned earlier, the high-temperature operation of YSZ is necessary for oxygen ion conductivity and to minimize the ohmic losses. In recent years, approaches making use of confinement effects at the nanoscale to improve device performance have emerged as an interesting alternative [13]. Decreasing the size of the material to the nanometer-length scale may lead to a dominant influence of the interface on the overall ionic conductivity of the system. In addition, heterostructures combining transition metal oxides can accommodate very large amounts of epitaxial strain without breaking into islands or structural domains, leading to enhanced ion diffusivity. A well-studied material combination is Y_2_O_3_-ZrO_2_/SrTiO_3_ (YSZ/STO) heterostructures where different structures (fluorite vs perovskite) are combined with a large lattice mismatch of 7%. The interface between highly dissimilar structures stabilizes a disordered oxygen sublattice with an increased number of oxygen vacancies, which, in turn, may promote oxygen diffusion even at a low temperature [14]. In the past, this YSZ/STO heterostructure has been used as a solid electrolyte in low-temperature fuel cell applications [15]. Inspired by the available literature, we have investigated the use of nanoscale heterostructures of YSZ/STO in the design of a mixed-potential gas sensor. The goal of this article is to study the heterostructure sensor response at reduced operating temperatures (around 200 °C). In this work, the low-temperature mixed-potential sensors were designed to detect hydrocarbons and NO_x_ in the open-circuit and biased modes, respectively. The electrode choice and the analyte selection were based on previous studies [16].

## 2. Experimental

Based on the mixed-potential sensor design developed earlier [17], ESL ElectroScience developed a High Temperature Co-fired Ceramic (HTCC) structure (both substrate tape and screen-printed patterns for the various layers) using well-established manufacturing methods, along with fully stabilized YSZ substrate tape. First, on the backside of the 1 mm thick, dense YSZ substrate tape, a Pt-heater with independent leads was printed to form the core of the new ceramic substrate. The Pt-heater was applied to a layer of insulating alumina tape to prevent ionic transport through the sensor body. A standard heater ink composition was used, and the required heater resistance was obtained by modifying the design of the heater pattern. An insulating overcoat was then applied to the Pt-heater to protect and reduce the amount of ambient heat loss, in order to provide a more uniform heat distribution across the substrate. Next, the sensing component, consisting of screen-printed La_0.8_Sr_0.2_CrO_3_ (MO) and Pt electrodes, was printed on the opposite side of the heater. Two special screens were used to print the electrodes. The MO and Pt electrode pads were made to a thickness of 500 nm so that the electrolyte completely covers the electrode and the leads were made to a thickness of 1 µm.

Lastly, the YSZ/STO thin-film heterostructure was sputter deposited through a shadow mask to cover and embed the MO and Pt electrodes. The YSZ and STO sputter targets (99.9% pure) were obtained from Kurt J. Lesker. First, a 50 nm thin film of STO was deposited at a Radio Frequency (RF) power of 150 W for 15 min, and then YSZ was deposited at a RF power of 200 W for 20 min to achieve a thickness of 100 nm. Throughout the sputtering process, the substrate was placed on a heated plate at 175 °C. The sputtering process was repeated to achieve 5 layers of STO (50 nm)/YSZ (100 nm). Finally, the device structure was sintered at 1100 °C in the presence of Argon. A control device with a plain YSZ thin film was fabricated to compare the performance of the sensors with and without the STO multilayer. The plain YSZ thin film was obtained by sputtering for a thickness of 750 nm matching the YSZ/STO multilayer.

The sintered thin films were then inspected using a Transmission Electron Microscope (TEM). TEM cross-sections of the multilayer films were prepared using focused ion beam (FIB)-based milling (FEI). A thin layer of Au was evaporated onto the multilayer film before FIB milling to protect the region of interest and to prevent charging. Coarse FIB milling was done at 30 kV, and the samples were then thinned to approximately 50 nm thickness at 2 kV. The samples were then examined in an aberration-corrected TEM operated at 300 kV. In the presence of air (partial oxygen pressure of 21%), the electrical conductivity of the YSZ/STO thin film was measured using AC impedance measurement on the Potentiostat (Princeton Applied Research PARSTAT 4000 potentiostat/galvanostat). The measurements were done in the impedance mode with a frequency sweep from 13 MHz down to 10 mHz and a perturbation of 10 mV at different temperatures. To compare the oxygen ion conductivity surrogate from the YSZ/STO multilayer and control YSZ thin film, the impedance spectrum was collected at 21% oxygen with a temperature reading of 200 °C and 25 ppm of propylene. A Keithley 2400 Source Measure Unit (SMU) was used to record the open-circuit sensor response. The SMU was connected to the fabricated sensor such that the Pt counter electrode was instrument positive and the metal oxide electrode was instrument negative.

A specially prepared quartz tube was used to test the fabricated thin-film sensor under the test gas stream using a method very similar to commercial automotive sensor testing. A commercial flat plate O_2_ sensor mount, using a standard configuration of four metal spring-loaded clips, was used to hold the fabricated sensor. A rubber stopper was used to hold the packaging in place and silicone gasket sealant was used to seal the gaps around the four wires providing electrical contact to the sensor electrodes and heater. The sensor was heated to 200 °C with a heater voltage of 4.5 V and a current of 0.2 A (0.9 W).

To get an accurate temperature reading of the devices, a P-L-type precision RTD sensor from Omega was placed in the tube furnace near the device. Air was used as the base gas. The flow rates of the various gas mixtures were controlled using an automated Trace Vapor Generator (TVG). The TVG was also used to generate 25 ppm of NO, NO_2_, NH_3_, C_3_H_6_, and CO. In this article, the fixed concentration of the gases was chosen to test the concept of low-temperature operation of the sensors. The low-temperature operation of sensors will enable low power consumption, which might be critical for applications such as air quality monitoring. However, the concentration range of analytes for such an application is in the order of parts per billion (ppb). These mixed-potential sensors will be engineered in future to sense ppb levels of pollutants or analytes.

The flow rates were fixed at 500 sccm. Two terminal measurements were performed at a fixed total gas flow rate and a fixed oxygen partial pressure of 21%. The rise time (t_90_, rise) and fall time (t_90_, fall) are defined as the time required for the sensor output to reach 90% of the full-scale value (maximum voltage) and as the time required for the sensor output to diminish to 10% of the full-scale value, respectively. The mixed-potential sensor response to an analyte was calculated as the voltage differential of the base gas (air) and the target/test gas.

## 3. Results and Discussion

**Figure 1:**
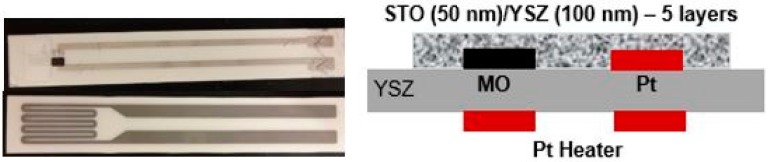
Mixed-potential sensing device and its cross-section image. (**Left**) Front and rear of the sensor showing the electrodes (Mo, Pt, and YSZ/STO electrolyte) and the Pt heater; (**Right**) Cross-section image showing the electrolyte embedding the electrodes.

Figure 1 shows the front, rear and cross-sectional image of the fabricated sensor. The embedded MO (black) and Pt (gray) electrodes, along with the YSZ/STO electrolyte (whitish strip), are clearly visible.

**Figure 2:**
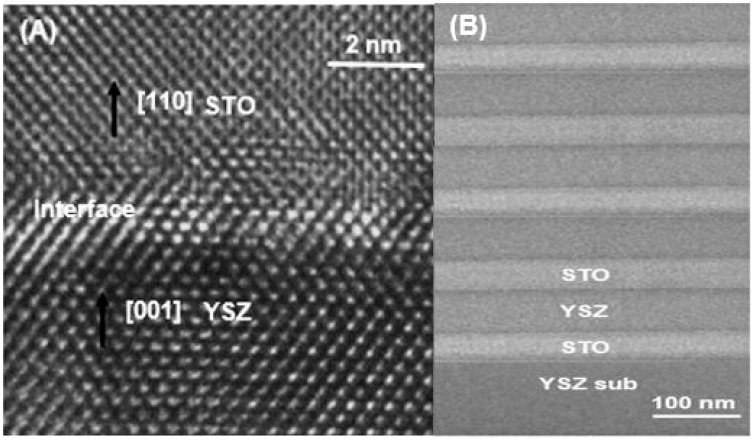
TEM micrograph of the device. (**A**) High-resolution TEM image YSZ/STO thin film, and the interface indicating good crystallinity of the sample; and (**B**) Low-magnification TEM image of cross-section showing the YSZ substrate, five layers of STO thin film (thickness around 50 nm)/YSZ thin film (thickness around 100 nm).

Figure 2 shows TEM micrographs of the YSZ/STO heterostructure. Figure 2a displays a representative high-resolution TEM image of the YSZ thin film, STO thin film, and the interface region showing the good crystalline quality of the sample aligned in the [001] and [110] direction. The YSZ/STO interface was seen to be largely flat and continuous with some slight distortion in the few atomic layers. The YSZ film showed good structural coherence with the STO crystal. From the TEM observations, an in-plane interfacial strain of 7.1% in the interface was calculated using the lattice constants of 3.65 Å and 3.91 Å for YSZ and STO layers respectively.

Figure 2b shows the low-magnification TEM image of the YSZ substrate, five layers of STO and the YSZ thin film after sintering. The interface between the substrate and the thin film and the interface between YSZ and STO thin films appear to be well-defined without any inter-diffusion after sintering.

**Figure 3:**
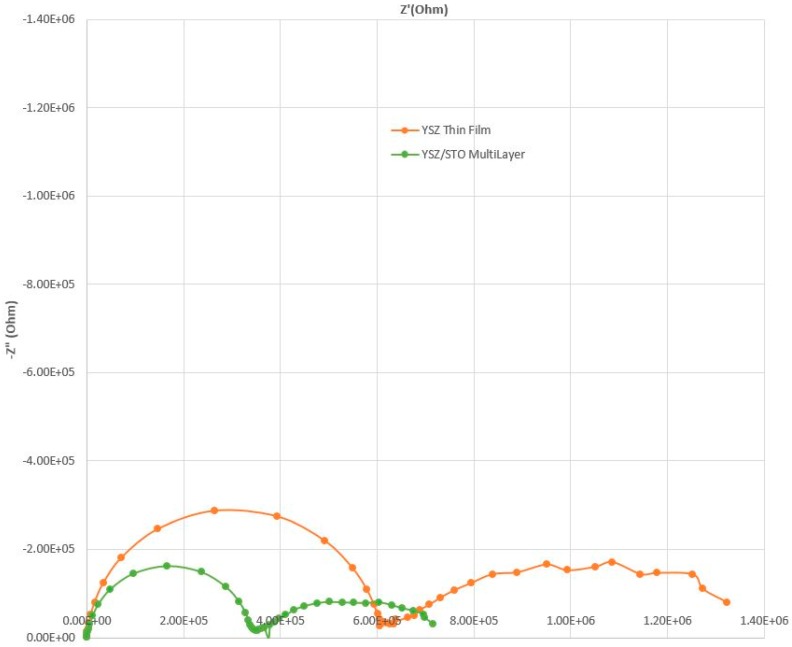
Comparison of impedance spectra obtained from two devices made of YSZ/STO multilayer thin-film electrolyte and plain YSZ thin film at 200 °C, 21% O_2_, and 25 ppm of C_3_H_6_.

The conductivity of the multilayer thin film was found to be thermally activated with an Arrhenius behavior. The activation energy for the YSZ/STO films was calculated to be 0.76 eV as opposed to 0.95 eV for plain YSZ film. The activation energy for oxygen ion conductivity was reduced for the YSZ/STO thin film, similar to the observation reported earlier [18]. Figure 3 shows the impedance spectrum comparison of devices with the YSZ/STO multilayer thin-film electrolyte and the plain thin-film YSZ electrolyte. In an impedance spectrum obtained from an electrochemical device, the contributions of the bulk electrolyte and the electrode/electrolyte interface are identified by the frequency dispersions. In general, the low-frequency region represents the electrode polarization/charge-transfer phenomenon while the high-frequency component can be approximated to ionic conduction of the electrolyte. The semi-circles at the high-frequency region indicate a lower resistance to oxygen conduction for the YSZ/STO-film-based device as compared with that of the plain YSZ thin film.

The interface in the planar oxide heterostructure film (YSZ/STO) seems to act as a fast oxygen ion conduction pathway. Oxygen vacancies and oxygen conduction arises from the interface strain and structural incompatibility between YSZ and STO. The MO/YSZ-STO/gas acts as the three-phase interface, otherwise known as the triple-phase boundary. Humidity was not added for this experiment.

**Figure 4:**
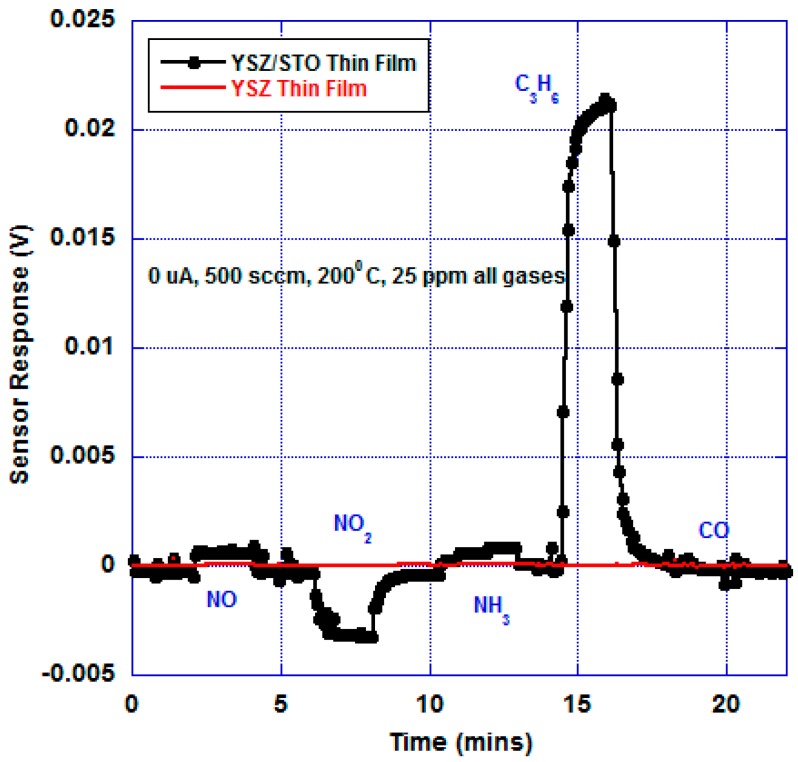
Open-circuit mixed-potential sensor response to various analytes (25 ppm) at 200 °C, 500 sccm, and oxygen partial pressure of 21%. The sensor made of YSZ/STO thin-film electrolyte showed a dominant C_3_H_6_ response, whereas the sensor made of just the YSZ thin film showed no response. The letter ‘u’ stands for the symbol ‘μ’. The baseline response is 0 V. Humidity was not added in this experiment.

Figure 4 shows the open-circuit mixed-potential response of the sensors in response to various analytes at 200 °C at an oxygen partial of 21%. The concentrations of all the analytes were maintained at 25 ppm. The sensor response of the device fabricated with just the YSZ thin film was compared with the response of the device with the multi-layer YSZ/STO film at 200 °C. The device with just the YSZ thin film did not show any discernible response to the analytes with a near-zero baseline response. However, the sensor device with the YSZ/STO thin film showed a dominant propylene response.

This sensor device responded to NO, NO_2_, and NH_3_. A slightly stronger signal to ammonia is due to direct oxidation of ammonia on YSZ/STO/Pt, where three ammonium ions are formed for every four ammonia molecules, in an overall three-electron electrochemical process. As expected, the sensor showed selective response to hydrocarbons. The sensor response and recovery time in response to C_3_H_6_ was found to be 65 and 70 s, respectively. The supplemental Figure S1 shows the open-circuit sensor response comparison to 25 ppm of C_3_H_6_ and a mixture of 25 ppm of C_3_H_6_, NO, NO_2_, and NH_3_. A 9.5% decrease in sensor response to C_3_H_6_ was observed when exposed to a complex mixture in comparison to isolated exposure of C_3_H_6_. The supplemental Figure S2 shows the sensitivity of the device as a function of different concentrations of C_3_H_6_.

A stable sensor response, even at such a low temperature (200 °C), indicates a strong oxygen ion conductivity which may be attributed to space charge effects, epitaxial strain, and atomic reconstruction at the interface of the YSZ/STO thin film [19]. The discontinuity of free energy at the interface may result in charge transfer processes that break charge neutrality and may profoundly change the carrier density. In ionic compounds, the accumulation of defects may create an electric field that can be screened over a space-charge layer by depletion or accumulation of mobile charges. The ionic interactions are found to be essential to reproduce the effective activation energy, and the enhanced oxygen ion mobility may result from a non-random distribution of dopant Y ions at the interfacial planes, structural disorder, or a decrease in ionic interactions when the layer thickness is down to the nanometer scale. We believe that the combination of epitaxial strain and oxygen sub-lattice incompatibility between the YSZ and STO structures may be the key in yielding high oxygen ion conductivity.

**Figure 5:**
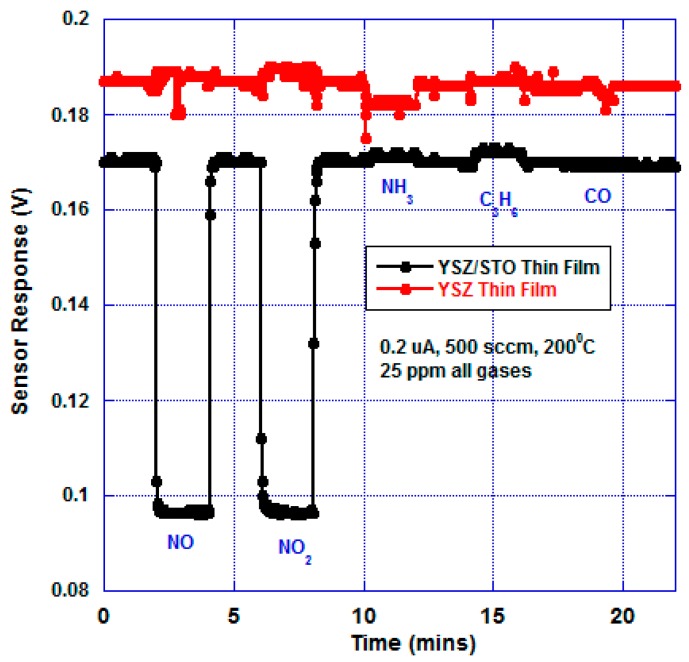
Current-biased mixed-potential sensor response to various analytes (25 ppm) at 200 °C, 500 sccm, and oxygen partial pressure of 21%. The sensor made with a YSZ/STO thin-film electrolyte showed selective NO_x_ response, whereas the sensor made of just the YSZ thin film showed no response. The letter ‘u’ stands for the symbol ‘μ’. Humidity was not added in this experiment.

Figure 5 shows the response of the sensor under bias. The bias current was chosen by trial and error in order to achieve selective NO_x_ response and equal magnitude responses for NO and NO_2_. The sensor device with just the YSZ thin film showed a different baseline compared with the YSZ/STO thin-film sensor. At 200 °C, there was no significant response to any analytes from the YSZ thin-film sensor whereas the sensor with the YSZ/STO electrolyte was NO_x_ selective. The supplemental Figure S3 shows the device sensitivity as a function of NO_2_ concentration. The sensor response and recovery time towards NO_x_ were found to be 7 and 8 s, respectively.

**Figure 6:**
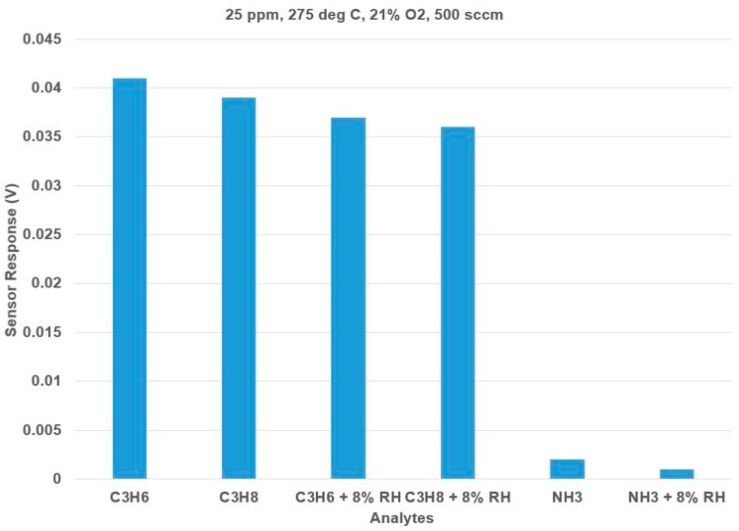
Effect of humidity (8% RH) on the sensor response to Hydrocarbons and Ammonia. The standard deviation after three trials of measurements is 0.002 V.

Next, the effect of humidity on C_3_H_6_, C_3_H_8_, and NH_3_ analytes were tested at 275 °C using a bubbler. Figure 6 shows the effect of humidity on the hydrocarbons and ammonia. With 8% RH, the sensor had a 9.7% decrease in the sensor response to hydrocarbons and ammonia. The dominant open-circuit sensor response to C_3_H_6_ is relatively unaffected by the addition of humidity.

Typically, the sensitivity of a mixed-potential sensor decreases with an increase in operating temperature. To identify the lower bound and upper bound of the sensor operating temperature, the sensitivity to hydrocarbons was monitored (figure not shown) as a function of temperature. Above 400 °C, the sensitivity to hydrocarbons drops. At 550 °C, the sensitivity drops close to zero, confirming the mixed-potential sensing behavior. On the other hand, below 175 °C, the sensitivity drops to 60% of the value at 200 °C. A detailed Gas Chromatography–Mass Spectrometry is needed to completely explain the effect of operating temperature on the sensor performance; this will form a part of future studies.

**Figure 7:**
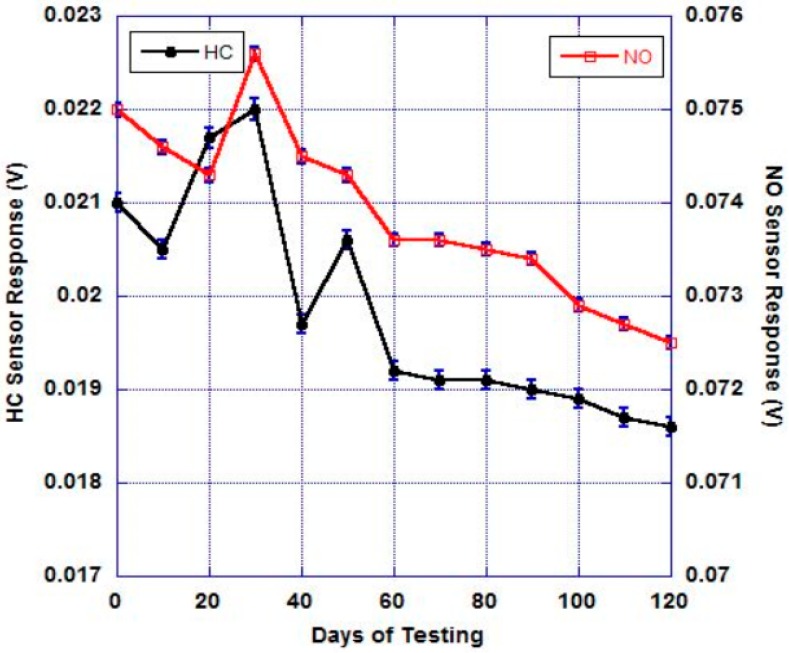
Long-term testing of the sensor (25 ppm, 200 °C, 500 sccm). A decrease in HC and NO mixed-potential sensitivity was observed after 120 days of testing. The error bars indicate three trials of analyte exposure. Humidity was not added in this experiment.

Next, the long-term stability (120 days) of the YSZ/STO thin-film sensor was evaluated at 200 °C (Figure 7). The sensor was tested every 10th day for HC and NO_x_ response by maintaining all the experimental conditions as constant. The sensor was kept in ambient air (oxygen partial pressure of 21%) and exposed to analytes (25 ppm) on alternate days (idle days) to identify any aging or sensor degradation over time.

After 120 days, the sensor showed slight decrease in HC and NO_x_ sensor response. An 11.4% decrease in HC response and a 3.3% decrease in NO_x_ response were observed.

The applications for low-temperature gas sensing with minimal power requirements has seen an escalated interest, particularly with the advent of the Internet of Things (IoT). The features of electrochemical sensors make them an ideal choice for IoT devices. The promising results reported in this article indicate that solid electrolytes such as YSZ/STO thin-film heterostructures can enable low-temperature and low-power gas sensors. Another application space for these sensors is air-quality monitoring.

Urban air quality represents a major public health burden and is a long-standing concern to citizens. Air pollution is associated with a range of diseases, symptoms, and conditions that impair health and quality of life. In particular, there is critical need to monitor NO and NO_2_ levels in ambient air. Short-term exposures (less than 3 h) to low levels of NO_2_ can lead to changes in airway responsiveness and lung function in individuals with preexisting respiratory illnesses. Further, long-term exposures to NO_2_ may lead to increased susceptibility to respiratory infection and may cause irreversible alterations in lung structure. Atmospheric transformation of NO_x_ can lead to the formation of ozone and nitrogen-bearing particles. Nitrogen oxides contribute to a wide range of effects on public welfare and the environment, including global warming and stratospheric ozone depletion. Deposition of nitrogen can also lead to fertilization, eutrophication, or acidification of terrestrial, wetland, and aquatic (e.g., fresh water bodies, estuaries, and coastal water) systems.

There is a current trend worldwide to increase the collection of air quality data beyond reference monitoring stations. Low-cost sensor platforms play a key role in air quality monitoring. Sensor nodes can be deployed as dense networks or mounted on vehicles, facilitating the elaboration of high-resolution air quality maps. Low-cost sensors with minimal power consumption, robust sensitivity, and selectivity against interferences with an adequate detection limit are the need of the hour. For example, the concentrations of NO_x_ in ambient air can vary from 1 ppb to 100 ppb. The sensor reported in the article can measure reliably up to 5 ppm with a reasonable power consumption. To improve the selectivity and detection limit, methods such as incorporation of nanomaterials in the electrodes and a new signal conditioning technique [20], referred to as the pulsed discharge technique, can be adopted.

## 4. Conclusions

In this article, a mixed-potential-based electrochemical gas sensor that can be operated at low temperatures (200 °C) was reported. The sensor was constructed on an YSZ substrate with metal oxide and Pt electrodes, along with an YSZ/STO multilayer solid electrolyte. The sensor exhibited a dominant hydrocarbon response at open circuit and a selective NO_x_ response under bias. The device constructed with just the YSZ thin film and electrodes showed no response at 200 °C, indicating an enhanced oxygen ion conductivity from the YSZ/STO heterostructure. After 120 days of testing, only an 11.4% decrease in HC response and a 3.3% decrease in NO_x_ response were observed. In addition, the fabricated sensor device only consumed less than a 1 W of power; for comparison, the plain YSZ thin-film device consumes about 5W of power under the standard operating temperature of 500 °C.

## Supplementary Materials

The Supplementary Materials are available online at http://www.mdpi.com/1424-8220/17/12/2759/s1.
