# Supplementary material for: A New Low-Temperature Electrochemical Hydrocarbon and NOx Sensor
Source: Sensors (Basel). 2017 Nov 29;17(12):2759. doi: 10.3390/s17122759 (PMC5750761; doi:10.3390/s17122759)
Supplement: Supplementary file 1 [file sensors-17-02759-s001.pdf]

Supplemental Information

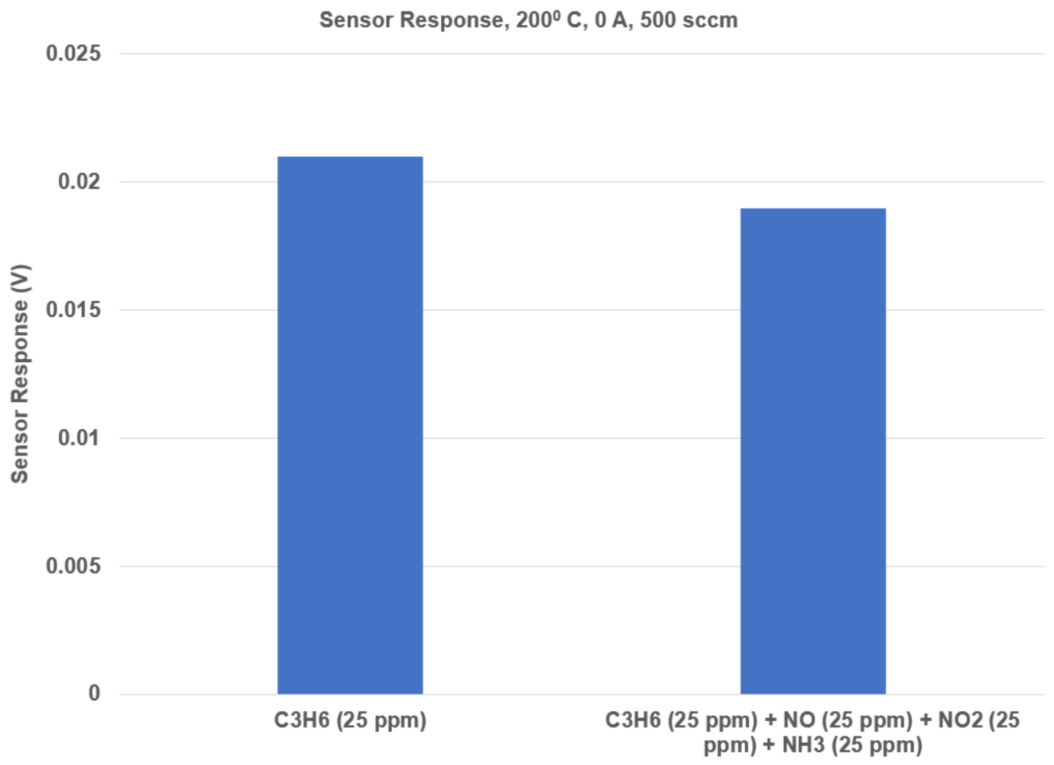

Sfigure 1. Open-circuit sensor response to 25 ppm of C_3H_6 and a mixture of 25 ppm of C_3H_6, NO, NO_2 and NH_3.

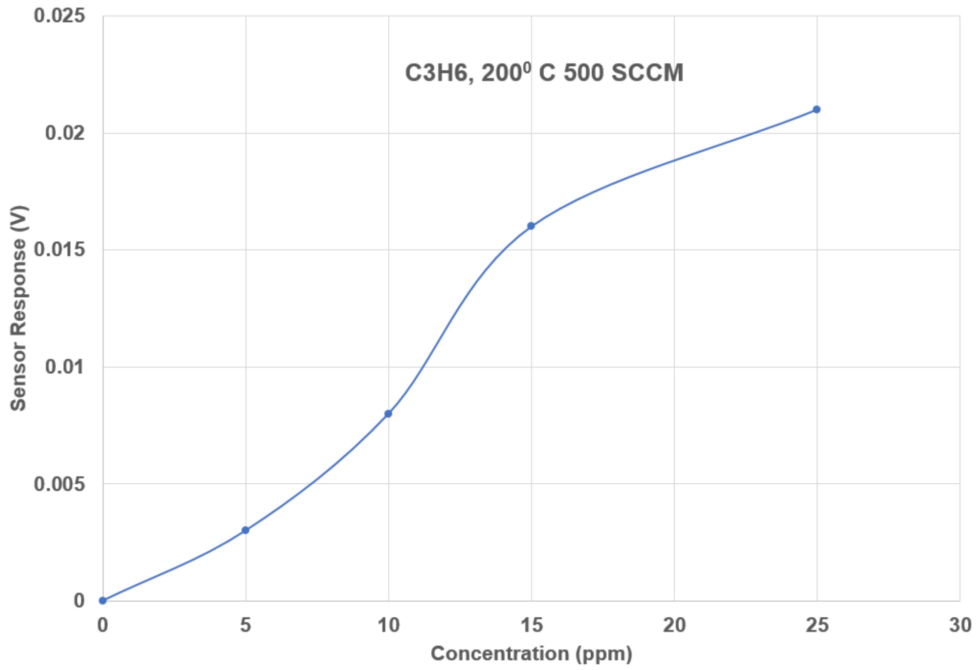

Sfigure 2. Open-circuit sensor response (sensitivity) to different concentrations of C_3H_6

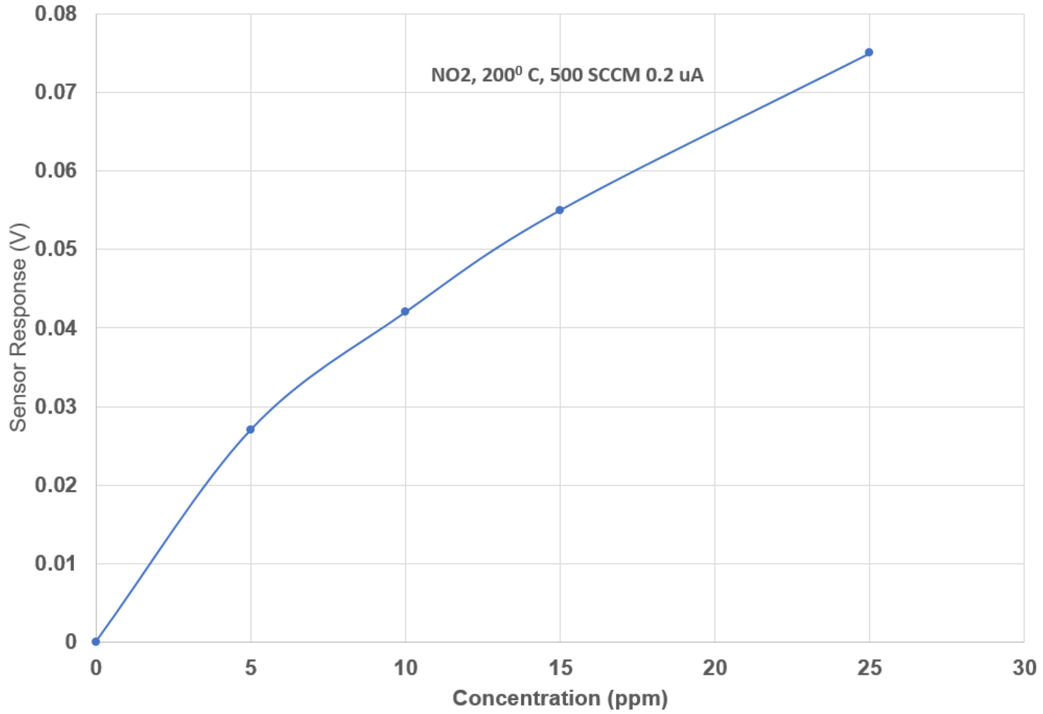

Sfigure 3. Biased sensor response (sensitivity) to different concentrations of NO_2
